# Developing an *In Vitro* Model of Endotoxemia to Assess the Immunomodulatory Effects of Anti-Inflammatory Peptide-Secreting Living Therapeutics

**DOI:** 10.1021/acsptsci.5c00216

**Published:** 2025-06-30

**Authors:** Ketaki Deshpande, Varun Sai Tadimarri, Juliette Ramirez-Rangel, Shrikrishnan Sankaran, Sara Trujillo

**Affiliations:** 1 INM - Leibniz Institute for New Materials, Saarland University, Campus D2 2, Saarbrücken 66123, Germany; 2 Saarland University, Saarbrücken 66123, Germany

**Keywords:** therapeutic peptides, immune response, cytokines, *Lactiplantibacillus plantarum*, probiotics

## Abstract

Living therapeutics are attractive candidates to tackle the limitations of classically delivered therapeutic peptides, which are often poorly stable and require cost-intensive modifications. Their functional assessment is limited to animal experiments, which increase the complexity to evaluate the dynamic nature of these systems. Therefore, we developed an *in vitro* model of endotoxemia using macrophages to assess early-stage anti-inflammatory Living therapeutics. We refined the model based on three anti-inflammatory peptides (KCF-18, I6P7, and α-MSH) and identified suitable therapeutic concentrations and treatment durations. We applied the model to *Lactiplantibacillus plantarum* TF103, a probiotic engineered to secrete these peptides. The model revealed that Living therapeutics enhanced the effects of the peptides, requiring lower amounts of anti-inflammatory effects. This points to potential synergistic effects between peptides and bacteria. The model presented here allows the investigation of dynamic regimes, which could be useful in the development of complex systems such as the ones encountered in Living therapeutics.

## Graphical abstract

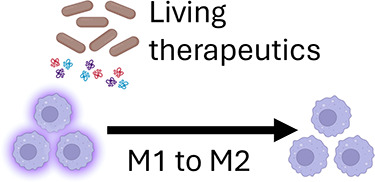

Therapeutic peptides are short amino acid chains that modulate biological processes by acting as hormones, enzymes, or signaling molecules. More than 100 peptides have been approved by the Food and Drug Administration worldwide in the past two decades.^1^ Most approved therapeutic peptides target chronic disorders such as diabetes (insulin) or obesity (semaglutide), which have a common symptom: inflammation.^2^ Thus, the development of anti-inflammatory peptides has increased.^3^ Some limitations of therapeutic peptides reside in their short half-life and low stability.^4^ To overcome this, therapeutic peptides are often modified with spacers such as polyethylene glycol, non-natural amino acids, cyclization loops, etc.,^1,5^ which increase manufacturing costs and can impact their efficacy.

Living therapeutics emerged as a promising technology to tackle this limitation by engineering microbes to freshly produce and secrete the therapeutics in the body.^6–10^ Probiotic and commensal bacteria have shown promise as Living therapeutics,^11^ currently in the pipeline as clinical candidates for different applications^12–19^ in particular to treat gut diseases and solid tumors.^20^ Lactic acid bacteria—both wild type and genetically engineered—are being developed to treat chronic disorders such as diabetic foot ulcers,^21^ wound healing,^22^ or stress.^23^ The results in safety and efficacy are promising. For example, two different studies showed no major toxicity or presence of their engineered lactic acid bacteria in distant organs when treating mice and mini pigs for wound healing applications.^21,22^ Inhaled *Lactobacilli* have also shown to reduce inflammation in bronchopulmonary dysplasia.^24^ However, with the increasing number of promising Living therapeutic candidates, it is necessary to streamline and design fundamental assays to assess these living systems, considering their complex and dynamic nature. For this, a solid method to evaluate their toxicity, potency, and mechanism of action is required. This is particularly challenging for microbes engineered with anti-inflammatory capabilities due to the added complexity and dynamism of immune cell responses. Typically, Living therapeutics are assessed directly in animal models, increasing the difficulty of investigating dynamic changes. Therefore, reliable *in vitro* models are needed to systematically assess the effects of Living therapeutics. This, together with the ethical desire to reduce animal use for experimentation, motivated this work.

Here, we report an *in vitro* model of endotoxemia using macrophages for the early-stage assessment of engineered microbes that secrete anti-inflammatory peptides. Development and optimization of the model were performed based on the activity of three previously reported therapeutic peptides with potential anti-inflammatory properties: KCF-18,^25^ I6P7,^26^ and α-melanocyte stimulating hormone (α-MSH).^27^ KCF-18 was designed to inhibit the binding of the pro-inflammatory cytokines tumor necrosis factor-α (TNF-α), interleukin-1β (IL-1β), and interleukin-6 (IL-6) with their receptors.^25,28,29^ I6P7 inhibits the binding between IL-6 and IL-6R by binding to IL-6R with high efficiency.^26,30,31^ α-MSH is an endogenous neuropeptide known for its pleiotropic functions (i.e., pigmentary, anti-inflammatory, antipyretic, and immunoregulatory roles).^32^ α-MSH inhibits acute and chronic inflammation in several tissues by binding the melanocortin 1 receptor (MC1R), also present on macrophages.^33^

Once optimized, the model was applied to test the activity of these peptides secreted by a probiotic lactic acid bacterium, *Lactiplantibacillus plantarum* TF103. We observed that the wild-type microbe had anti-inflammatory effects, which increased the effects observed for the secreted peptides by the bacteria, even at lower concentrations.

## Experimental Section

### Anti-Inflammatory Peptides

Recombinant peptide KCF-18 (purity 95%, sequence: KCRKEMFKQKLPYSTVYF) (GeneCust) was diluted in Milli-Q water at a concentration of 0.5 mg/mL and used as a stock (stored at −20 °C). Recombinant peptide I6P7 (purity 95%, sequence: LSLITRLGE) (GeneCust) was diluted in Milli-Q water, dimethyl sulfoxide (DMSO), and ethanol in a 2:1:1 ratio at 0.5 mg/mL (stock, stored at −20 °C). Recombinant peptide α-MSH (purity 95%, sequence: SYSMEHFRWGKPV) (GeneCust) was diluted in Milli-Q water at 512 μg/mL (stock, stored at −20 °C).

### Cell Culture

The murine macrophage cell line J774-Dual (InvivoGen, passages 3–8) was cultivated in a growth medium consisting of Dulbecco’s modified Eagle’s medium (DMEM; Gibco), containing 10% heat-inactivated fetal bovine serum (PAN Biotech) and supplemented with 100 U/mL penicillin, 100 μg/mL streptomycin (Gibco) and 100 μg/mL Normocin (InvivoGen) at 37 °C in a 5% CO_2_ incubator. The growth medium was exchanged every other day, and the cells were subcultured after growing to 70–90% confluency. J774-Dual cells express a secreted embryonic alkaline phosphatase (SEAP) reporter gene under the control of an IFN-β minimal promoter fused to the NF-κB consensus transcriptional response element and the c-Rel binding site. They also express the Lucia luciferase gene, encoding a secreted luciferase under the control of an ISG54 minimal promoter, together with the IFN-stimulated response element.

### Cytocompatibility Assays

Peptide cytotoxicity was carried out via a lactate dehydrogenase assay (LDH Assay, CytoTox 96 Nonradiative Cytotoxicity Assay, Promega) following manufacturer instructions. Briefly 10,000 cells/well were seeded in μ-Plate Angiogenesis 96-well plates (Ibidi) in growth medium (70 μL) and allowed to attach overnight (16 h, 37 °C, 5% CO_2_). Then, the medium was removed, and cells were washed twice with phosphate buffer saline (PBS, without calcium and magnesium, VWR) before being treated with different concentrations of anti-inflammatory peptides (Table S1) and incubated overnight (37 °C, 5% CO_2_). Then, 30 μL of supernatants from each well was transferred to a new 96-well plate, and 30 μL of LDH substrate was added. Samples were incubated in the dark for 30 min at room temperature. Then, 30 μL of the stop solution was added, and the absorbance at 490 nm was read using a plate reader (TECAN Spark). Negative control (cells in growth medium), lysis control (cells treated with 37.5% Triton X-100 for 1 h), and blanks (pure anti-inflammatory peptides in growth medium) were also analyzed. The percentage of cell death was calculated as

$$\mathrm{cell}\;\mathrm{death}\left(\%\right)=\left[{\mathrm{sample}}_{\mathrm{absorbance}}-\mathrm{blank})/\left({\mathrm{lysis}}_{\mathrm{absorbance}}-\mathrm{blank}\right)\right]\times 100$$

Cell viability after peptide treatment was also quantified via the alamarBlue assay (Invitrogen) following the manufacturer’s guidelines. Briefly, 70 μL of alamarBlue reagent (10% v/v in growth medium) was added to treated cells (cells incubated for 24 h with pure anti-inflammatory peptides) and incubated for 2 h. The supernatant was then transferred to black 96-well plates. Fluorescence (Ex/Em 570/600 nm) was measured by using a plate reader. Negative control (cells in growth medium) and blanks (10% alamarBlue reagent in growth medium) were also analyzed. Fluorescence values were normalized with the blanks.

### Macrophage Stimulation

Macrophages were seeded in 96-well plates at a density of 5 × 10^4^ in 200 μL of growth medium and incubated overnight (37 °C, 5% CO_2_). For experiments with the human monocytic leukemia cell line, THP-1 (ATCC, Germany) (passages 3–5), cells were seeded in 96-well plates at a density of 5 × 10^4^ in 200 μL of growth medium. THP-1 monocytes were differentiated into macrophages by treatment with 150 nM 12-*O*-tetradecanoylphorbol-13-acetate^34^ (TPA, Sigma-Aldrich, Germany) and incubated for 48 h (37 °C, 5% CO_2_). To perform the pretreatment, 100 μL of the peptides at varying concentrations was added to the macrophages 1 or 2 h before the experiment. Following this pretreatment step, cells were stimulated with 100 ng/mL lipopolysaccharides (LPS) for an additional 2 h to induce an inflammatory response. After stimulation, the peptides were added for 24 h more to assess the inhibitory effects on inflammation under the stimulated conditions. The supernatants were collected the next day to evaluate the immune responses.

### Assessment of Immune Responses

#### SEAP Assay

SEAP expression under the NF-κB complex activity was assessed. The activity of the SEAP from the macrophages was assessed by following manufacturer instructions. Briefly, 180 μL of SEAP detection reagent (QUANTI-BLUE Solution) was added to the 20 μL cell culture supernatants from each well (samples), controls (stimulated with LPS only and untreated), and blank (containing only SEAP detection reagent) to a new 96 well-plate. Absorbance was measured at 655 nm by using a plate reader. Data was recorded and normalized with the blanks.

#### Luciferase Assay

Luciferase expression was assessed via luminescence quantification following manufacturer instructions. Briefly, 20 μL of cell culture supernatant from each well (samples), controls (stimulated with LPS only and untreated), and blank (containing only Lucia and Gaussia luciferase detection reagent) were transferred to a new white 96 well-plate, followed by addition of 50 μL of Lucia and Gaussia luciferase detection reagent (QUANTI-Luc 4 Lucia/Gaussia), and luminescence was immediately measured at 100 ms integration time using a plate reader. Data were recorded as relative light units (RLUs) and normalized with blanks.

#### Enzyme-Linked Immunosorbent Assay (ELISA)

IL-6 and TNF-α were quantified using sandwich ELISA (R&D Systems) following the manufacturer’s instructions. Briefly, 100 μL of cell culture supernatants from each well (samples), controls (stimulated with LPS only and untreated), and standards (either IL-6 or TNF-α) were incubated in a precoated ELISA 96 well-plates, followed by the addition of detection antibodies, streptavidin-HRP, and substrate reagents. Plates were washed thrice with wash buffer (0.05% Tween 20 in PBS) after every step. Absorbance was measured at 450 nm with wavelength correction at 540 nm using a plate reader (TECAN Spark). Cytokine concentrations were calculated by extrapolating a standard curve following a 4-parameter logistic curve.

#### Cytokine Array

Cytokine profiles were measured using the Cytokine Array Panel A (R&D Systems) following the manufacturer’s instructions. Briefly, 1 mL of the supernatants was collected and incubated with the array membranes preblocked in the manufacturer’s blocking buffer, including addition of biotinylated detection antibody cocktail and streptavidin (IRYDye 800CW, LI-COR) followed by chemiluminescence detection using an Odyssey M imager (LI-COR). Signal intensity for each cytokine was quantified using Empiria Studio Software v3.0.0.173, and the data were normalized with negative control for comparison.

#### Immunostaining

Cells were fixed with 4% paraformaldehyde (in PBS) for 20 min at room temperature and washed twice with PBS. Then, cells were permeabilized with 0.1% Triton X-100 (in PBS) for 5 min. After permeabilization, samples were washed twice with PBS and blocked by using blocking buffer (1% bovine serum albumin in PBS). After blocking, cells were incubated overnight at 4 °C with an anti-CD206 rabbit antibody (dilution 1:300, Invitrogen, Germany). The following day, cells were washed thrice with PBS, and the secondary antibody alexaFluor 594-conjugated donkey-antirabbit (dilution 1:400, Invitrogen, Germany) in a blocking buffer was added. For actin and nuclei staining, cells were incubated with CoraLite Plus 488-conjugated Phalloidin (dilution 1:400, Chromotek, Germany) and 4′,6-diamidino-2-phenylindole (DAPI) (dilution 1:1000, Invitrogen, Germany) for 1 h at room temperature protected from light. After staining, samples were washed five times with PBS, and cells were incubated in PBS while imaging. Images were taken using a Leica DMI6000 B epifluorescence microscope.

### Image Analysis

Image analysis was carried out using ImageJ software (version 1.53k). Images taken from immunostained samples of CD206, actin, and nucleus were used. Nuclei images from DAPI staining were used to quantify the number of cells per field of view (FoV). Images were converted to 8-bit, and the Blur filter was passed with a ball radius of 2. Then, the Find maxima tool was applied with a noise tolerance of 5, and the number of cells was obtained as the number of maxima points per image. Images from CD206 staining were converted to 8-bit, and the Blur filter was applied to the images. Then, the Find maxima tool was used with a noise tolerance of 10, and the number of CD206 positive cells was obtained (*n* = 10 images, conditions were assayed in triplicates). The percentage of CD206 positive cells was calculated as

$${\mathrm{CD}206}^{+}\;\left(\%\right)=\left({\mathrm{CD}206}^{+}\;\mathrm{cells}/\mathrm{total}\;\mathrm{number}\;\mathrm{of}\;\mathrm{cells}\right)\times 100$$

### Probiotic Genetic Modification

#### Strain, Media, and Plasmids

The *L. plantarum* TF103 strain was used.^35^ This is a derivative of the *L. plantarum* WCFS1 strain, in which genes associated with lactic acid production have been knocked out. De Man, Rogosa, and Sharpe (MRS) medium (Carl Roth) was used to grow the strain. The engineered *L. plantarum* TF103 strains were grown in MRS media supplemented with 10 μg/mL erythromycin (Carl Roth) at 37 °C and 250 rpm for 16–20 h.

#### Molecular Cloning Reagents

The following enzymes and kits were purchased from New England Biolabs: Q5 High Fidelity 2X Master Mix for PCRs, NEBuilder HiFi DNA Assembly Cloning Kit for Gibson Assembly, Quick Blunting Kit for DNA phosphorylation, and the T4 DNA Ligase enzyme for DNA ligation. DNA oligomers were purchased from Eurofins Genomics. Synthetic genes were synthesized as eBlocks from Integrated DNA Technologies (IDT) and codon-optimized using the IDT Codon Optimization Tool. DNA was extracted using a plasmid extraction kit (Qiagen). The DNA purification kit was purchased from Promega.

#### *L. plantarum* TF103 Competent Cell Preparation and DNA Transformation

*L. plantarum* TF103 wild-type cells (WT) were inoculated in 5 mL of MRS media without antibiotic and grown overnight at 37 °C (static). The next day, 1 mL of the overnight bacterial culture was transferred to a secondary culture with 32 mL of MRS and 8 mL of 1% (w/v) glycine. The secondary culture was incubated at 37 °C (static) until the optical density measured at 600 nm (OD_600_) reached 0.6–0.8. Cells were pelleted by centrifugation (4000 rpm, 10 min, 4 °C). Next, the pellet was washed several times by centrifugation (8 min, 4000 rpm). The first two washes were done with 5 mL of ice-cold 10 mM MgCl_2_. The next two washes were performed with 5 mL of ice-cold Sac/Gly solution [10% (v/v) glycerol and 1 M sucrose mixed in a 1:1 (v/v) ratio]. Last, after discarding the supernatant, the pellet was resuspended in 600 μL of Sac/Gly solution, and the competent cells were distributed in 60 μL aliquots for DNA transformation. For the transformation, 1–2 μg of dsDNA was added to 60 μL of competent cells and incubated on ice for 10 min. The mixture was transferred to ice-cold 2 mm gap electroporation cuvettes (Bio-Rad), and cells were electroporated with a single pulse at 1.8 kV, after which 1 mL of MRS media was immediately added. The mixture was then incubated for 4 h (37 °C and 250 rpm). After that, cells were centrifuged (4000 rpm, 5 min), and 800 μL of the supernatant was discarded. The remaining 200 μL was used to resuspend the pellet, which was plated on MRS Agar supplemented with 10 μg/mL erythromycin. The plates were incubated at 37 °C for 2–4 days to allow the growth of the bacterial colonies.

### Direct Cloning in *L. plantarum* TF103

Plasmid engineering of *L. plantarum* TF103 was done using the direct cloning method previously developed by us, which involved PCR-based amplification and circularization of recombinant plasmids, which were then transformed into the bacteria by electroporation.^36^ In brief, complementary overhangs for HiFi Assembly were either synthesized as custom-designed eBlocks or generated by PCR. The HiFi DNA Assembly reaction was performed following the manufacturer’s protocol. Then, 5 μL of the assembled HiFi product was used as a DNA template in the PCR reaction (100 μL of final volume). After purifying the PCR product, 1000–2000 ng of linear DNA was phosphorylated using the Quick Blunting Kit following the manufacturer’s protocol. Phosphorylated products were ligated with the T4 ligase enzyme. Two ligation reactions were set per cloning, each based on 1000 ng of phosphorylated DNA, 2.5 μL of 10× T4 Ligase Buffer, and 1.5 μL of T4 Ligase enzyme (Milli-Q water was added up to 25 μL). The ligations were incubated at 25 °C for 8 h and then at 70 °C for 10 min for enzyme inactivation. After that, the ligations were mixed and purified, performing three rounds of elution to concentrate the DNA (each based on 10 μL of Milli-Q water). The entire eluted mix (2000 ng) was transformed into *L. plantarum* TF103 electrocompetent cells.

### Bacterial Culture

Overnight grown *L. plantarum* TF103’s culture was subcultured to OD600 0.1 in MRS medium supplemented with erythromycin and incubated at 37 °C for 4–5 h until OD600 reached 0.6–0.8. Bacterial culture was spun down at 4000 rpm for 3 min, and the entire bacterial population was transferred to DMEM supplemented with erythromycin and incubated at 37 °C for 24 h. The cell free supernatant was collected and filter-sterilized using a 0.22 μm filter prior to treatment with macrophages.

### Split Nano Luciferase Assay

To measure the amount of fusion protein with the SmBiT tag, 25 μL of culture or culture supernatant was transferred to a white, flat-bottomed 96-well plate and 25 μL of 1 μM LgBiT in PBS was added. The furimazine substrate (Promega) was diluted 1:50 in Nano-Glo Luciferase assay buffer (Promega) and immediately added to the wells with SmBiT-LgBiT to a final volume of 100 μL. Plates were protected from light for 10 min at room temperature. Luminescence values were measured with an integration time of 100 ms (InfinitePro Tecan). Raw data was correlated with the standard curve obtained using the synthesized SmBiT peptide and purified LgBiT fragment to calculate the concentration of secreted proteins.

### Statistical Analysis

Statistical analyses were carried out with GraphPad Prism 7.0 software. All graphs represent mean ± standard deviation (SD). Samples were assayed in triplicate unless otherwise noticed. Experiments were carried out twice unless otherwise noted. For the cell experiments, the goodness of fit of the data was tested via the Normality Shapiro–Wilk test. Normal distributed populations were analyzed using the analysis of variance (ANOVA) test followed by Tukey’s *post hoc* test to correct for multiple comparisons. Non-Gaussian populations were analyzed via the nonparametric Kruskal–Wallis test followed by Dunn’s *post hoc* test to correct for multiple comparisons. Differences among groups are indicated as *p*-values <0.05 (*), *p*-values <0.01 (**), *p*-values <0.005 (***), *p*-values <0.001 (****), and differences among groups not statistically significant (ns).

## Results and Discussion

### Generation of an *In Vitro* Model of Endotoxemia Using Macrophages

We used the murine cell line J774-Dual macrophages to standardize our anti-inflammatory assays. This cell line has been previously reported to investigate anti-inflammatory activity of several compounds.^37–39^ J774-Dual expresses two reporters (SEAP and luciferase) under the regulation of nuclear factor-κB (NF-κB) and interferon regulatory factor (IRF), respectively. We used a treatment with lipopolysaccharide (LPS) to model endotoxemia, whose hallmark is high levels of circulating LPS in the body. LPS stimulates macrophages toward the M1 phenotype (pro-inflammatory) by binding to the Toll-like receptor-4 on the surface of macrophages, activating the inhibitory-κB kinase, the mitogen-activated protein kinase (MAPK), and the IRF3-mediated pathways, leading to the nuclear translocation of NF-κB, AP-1, IRF3, and STAT1 transcription complexes.^40^ This triggers a cascade of downstream signals characterized by the production of proinflammatory cytokines such as IL-6, IL-1, and TNF-α, among others (Figure 1a). These cytokines bind to their receptors, triggering the production of more proinflammatory signals.^41^ The response of macrophages to LPS varies depending on concentration and time.^42,43^ We first investigated the effect of LPS at different concentrations and treatment durations (Figure S1a). LPS concentration used to induce macrophages *in vitro* varies depending on the study (0.0025–1000 ng/mL).^44,45^ LPS (31.25–2000 ng/mL) was applied for 24 h and NO production, which is characteristic of M1 phenotype was measured by Griess assay.^46^ Cells were activated at a similar level. Concentrations between 62.5 and 125 ng/mL of LPS were enough to activate the macrophages, in agreement with others that reported the use of 100 ng/mL.^43,47^ We also tracked NO production over time (24, 48, and 72 h), as some studies report macrophage tolerance to LPS when exposed for longer times.^44^ We observed a decay in NO production at 48 and 72 h (Figure S1b). Therefore, we concluded that 24 h was the maximum exposure time for our system before cells desensitize from LPS. We also tracked the expression of NF-κB via the SEAP assay during the first hours after LPS treatment and observed that NF-κB activation peaked at 2 h post stimulation, after which the expression decreased slightly (Figure S1c). This was in accordance with reports by others.^48–50^ Based on this, we treated the cells for 2 h with LPS to stimulate the cells. We also incorporated lauric acid and dexamethasone as positive controls (LA; 300 μM, and DM; 5 μM) due to their known anti-inflammatory properties.^48,49^

**Figure 1:**
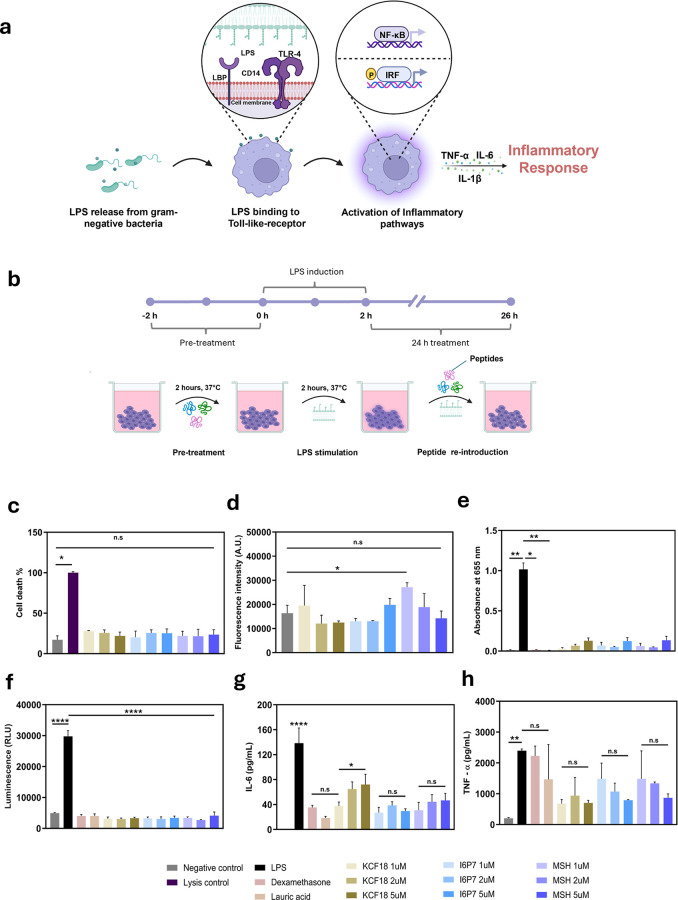
Anti-inflammatory effects of pure peptides on J774-Dual cells. (a) Schematic of inflammatory response of macrophages. (b) Sketch of the treatment protocol for the *in vitro* model of endotoxemia. (c) Percentage of cell death quantified by the LDH assay. (d) Fluorescence intensity of tetrazolium-metabolizing cells via the alamarBlue assay. (e) Absorbance measured at 655 nm for the NF-κB expression reporter assay via an alkaline phosphatase assay (SEAP assay). (f) Luminescence measurement for the IRF expression reporter assay via the luciferase assay. (g) Quantification of IL-6 produced by macrophages via ELISA (**** on top of LPS condition indicates comparisons to all other conditions). (h) Quantification of TNF-α produced by macrophages via ELISA. All graphs show the following: mean ± SD, *n* = 3. Statistically significant comparisons in the ANOVA test are highlighted as * (*p*-value <0.05), ** (*p*-value <0.01), *** (*p*-value <0.001), **** (*p*-value <0.0001), and n.s (not statistically significant).

We assessed the potential cytotoxicity of the anti-inflammatory peptides KCF-18, I6P7, and α-MSH at increasing concentrations. We observed no toxic effects up to 100 μM for I6P7 and KCF-18 and 800 nM for α-MSH (Figure S2). We then proceeded to treat our model of endotoxemia with different amounts of peptides for 24 h (after LPS stimulation; Figure S3a). We quantified the expression of NF-κB activation and IL-6 secretion (Figure S3). We observed that all peptide treatments had similar NF-κB activation compared to LPS (or higher). The quantification of IL-6 secretion revealed that the highest concentrations of the peptide had a slight effect on the reduction of IL-6 production (Figure S3e–g). KCF-18 acts as an antagonist of the IL-6-IL-6R, IL-1/IL-1R, and TNF-α/TNFR binding sites, and I6P7 is an antagonist of the IL-6R.^25,31^ α-MSH binds to the MC1R present in macrophages, which downregulates the expression of proinflammatory cytokines such as IL-6.^51^ We hypothesized that a pretreatment with the peptides would help block the receptors present on macrophages, so when the proinflammatory cytokines are produced after LPS treatment, the receptors that bind those cytokines are already blocked, stopping the proinflammatory positive feedback loop.^25^ For α-MSH, pretreatment was hypothesized that would enhance its effects, as binding the MC1R would also upregulate the transcription of anti-inflammatory pathways. We pretreated the macrophages for 1 h with different concentrations of the peptides and quantified NF-κB activation and IL-6 secretion (Figure S4). We observed that NF-κB was activated similarly to LPS for all treatment peptides and IL-6 secretion was slightly higher than the LPS control, meaning that there was no effect observed. We then investigated 2 h of pretreatment with peptides, which seemed to increase the anti-inflammatory effects on the macrophages (Figure 1).^52^ To make sure that 2 h of pretreatment followed by 24 h of treatment with the peptides was required to observe anti-inflammatory effects, we run an experiment where a 2 h pretreatment was carried out without addition of peptides after LPS stimulation (Figure S5). We observed that NF-κB activation was similar to peptide pretreatment to LPS control.

### Effects of KCF-18, I6P7, and α-MSH on the *In Vitro* Model of Endotoxemia

We investigated the effects of 1, 2, and 5 μM KCF-18, I6P7, and α-MSH with our *in vitro* model for endotoxemia (Figure 1). Cytotoxicity was assessed with the 2 h pretreatment followed by 24 h of treatment at these concentrations (Figure 1c). As expected, the additional treatment step did not trigger a higher cell death compared to previous experiments (Figure S2). The metabolic activity of the macrophages was investigated as well via the alamarBlue assay. No differences in cell metabolic activity were observed. We then looked at NF-κB and IRF activation.^53,54^ Figure 1e,f shows a reduction of NF-κB and IRF activation for all conditions at a level similar to the negative control (no LPS treatment), while LPS treatment showed the highest values. When looking at the production levels of IL-6 (Figure 1g), we observed a reduction in IL-6 production for all peptides at levels similar to those of the two positive controls, dexamethasone and lauric acid. We also measured the TNF-α production (Figure 1h). First, we observed that dexamethasone and lauric acid were not effective in reducing the production of the cytokine. Treatment with the peptides slightly reduced the production of TNF-α, where higher concentrations showed a higher inhibition effect. This could be due to the faster kinetics of TNF-α expression compared to IL-6 where TNF-α is produced earlier on and peaking at higher levels compared to IL-6,^55^ which might affect the inhibition effects of the peptides observed in our model.

Once we confirmed that our *in vitro* model of endotoxemia could be used to assess differential performance of the peptides (Figure 1), we used it to screen over 30 cytokines to gain more insights into the peptides’ immunomodulatory effects (Figure 2). We looked at the production of proinflammatory and anti-inflammatory cytokines. Results were normalized to those of the negative control. Some known anti-inflammatory cytokines such as IL-2, IL-1ra, IL-4, or IL-3 were upregulated compared to negative control and LPS-induced control. Some known proinflammatory cytokines were downregulated such as IL-1β, IL-6, or TNF-α (for KCF-18 treatment). Other potent chemoattractant proteins such as RANTES, G-CSF, or Eotaxin were expressed at high levels; however, their relative expression compared to LPS-treatment was reduced, indicating that the peptides might also influence these chemokines at a lower extent. As we observed an upregulation of classically anti-inflammatory (M2) phenotypes such as IL-4 and IL-1ra, we sought to quantify the percentage of M2 macrophages via immunostaining of the mannose receptor (CD206)^56^ (Figure 3). CD206 positive macrophages exhibit anti-inflammatory characteristics.^57^ First, we observed that the number of cells quantified was similar for all conditions. The highest expression of the mannose receptor was observed for the controls treated with dexamethasone and lauric acid (∼70%). It is noteworthy that KCF-18, I6P7, and α-MSH presented between 20 and 40% of M2 positive macrophages, indicating a shift in phenotype from proinflammatory to anti-inflammatory.

**Figure 2:**
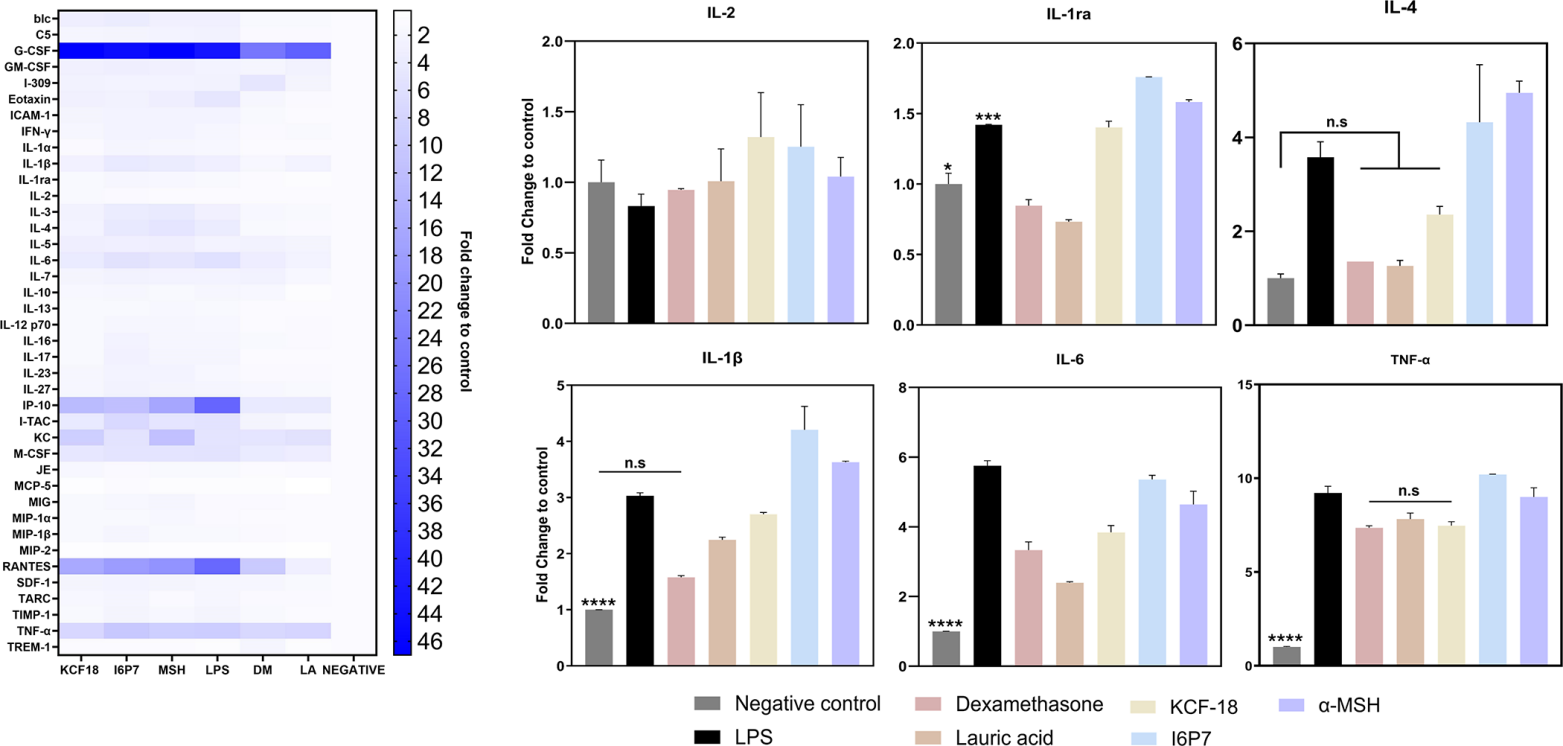
Macrophage cytokine profile changes after peptide treatment. Left: heatmap of the cytokine array, where 5 μM of KCF-18, I6P7, and α-MSH were used to treat LPS-stimulated macrophages (cytokine production is normalized to negative control, fold-change to control). Positive controls were dexamethasone (DM) and lauric acid (LA). Right: graphs from the cytokine array showing in detail changes in the secretion of selected cytokines (IL-2, IL-1ra, IL-4, IL-1β, IL6, and TNF-α).

**Figure 3:**
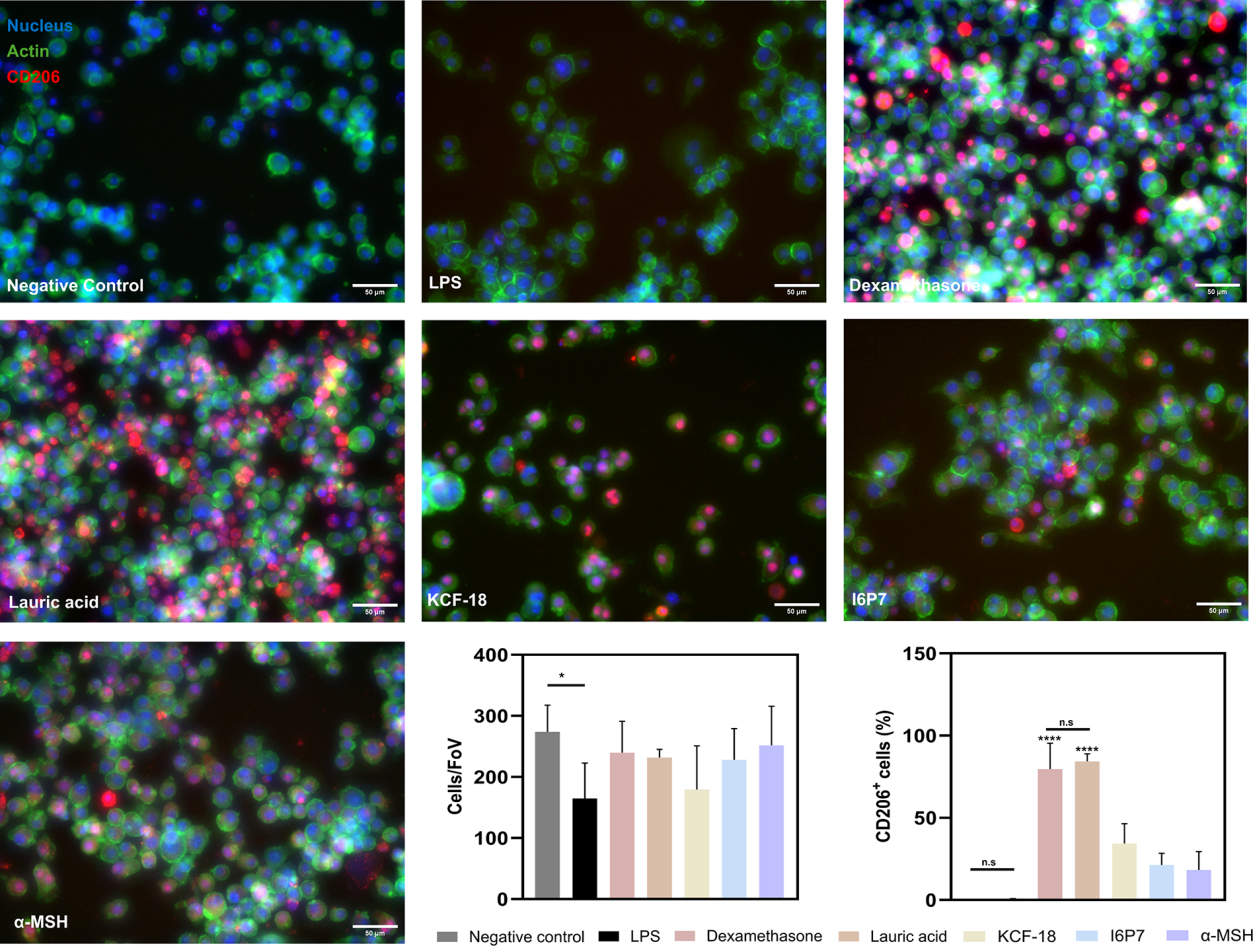
M2 macrophages quantified as CD206+ cells. Macrophages were treated with 1 μM of KCF-18, I6P7, or α-MSH and controls were LPS-treatment, dexamethasone-treatment, lauric acid-treatment, or no treatment (negative control) (scale bar 50 μm, blue: nuclei, green: actin, red: CD206). Bottom right graph: percentage of CD206+ cells quantified from the images (mean ± SD, *n* = 10 images/condition); bottom left graph: number of cells per field of view (FoV) quantified from the images (mean ± SD, *n* = 10 images/condition).

### Engineering *L. plantarum* TF103 to Secrete KCF-18, I6P7, or α-MSH Peptides

Encouraged by the previous results indicating that our *in vitro* model of endotoxemia can be used to assess the anti-inflammatory activity of therapeutic peptides, we applied it to assess a Living therapeutic scenario. We engineered *L. plantarum*, a probiotic that is being extensively explored for Living therapeutic applications,^9,11,58,59^ to secrete all three therapeutic proteins. We first tested the most extensively engineered strain, *L. plantarum* WCFS1, but found that it lowered the pH of the macrophage medium to ∼5 in 24 h, which was unsuitable for the subsequent culture of the macrophages. This drop in pH was most likely caused by lactic acid production, so we proceeded with a derivative strain, *L. plantarum* TF103, in which genes related to lactic acid metabolism have been knocked out.^35^ This resulted in no acidification of the medium after 24 h of incubation and demonstrated the potential use of this strain as a Living therapeutic candidate. This reduction in local acidification could improve host response to treatment with the strain, as extracellular acidification is considered a hallmark of inflammatory response.^60^ Knocking out genes that could trigger immune response is a strategy currently pursued in the field; for example, a human lung pathogen (*Mycoplasma pneumoniae*) was engineered to hinder gene transfer to most bacterial strains and genes encoding pathogenic factors were removed. The genome-reduced strain was further engineered to produce antibiofilm and bactericidal enzymes, which proved successful in eliminating *Staphylococcus aureus* biofilms *in vitro* and *in vivo*.^61^ Other strategies to improve tolerability by the host to live biotherapeutics involve the use of biomaterials to encapsulate the live biotherapeutic to avoid direct contact with the host.^62^

We engineered *L. plantarum* TF103 to secrete all three therapeutic peptides using genetic parts previously reported in *L. plantarum* WCFS1.^11^ Expression was driven by the strongest constitutive promoter, *P*_*tec*_, and secretion was mediated by efficient signal peptides, SP3 or 3050 (Figure 4a). The peptides were flanked at their N-terminals by the small fragment (SmBiT) of a split NanoLuc Luciferase protein, which is used for quantification of secretion when combined with its complementary large fragment (LgBiT). When the peptide is secreted into the medium, we can detect and quantify it by adding LgBiT to restore the split luciferase and measure the luminescence signal by adding furimazine as a substrate (Figure 4b). In total, we engineered three strains: *L. plantarum* TF103-secreting KCF-18, *L. plantarum* TF103-secreting I6P7, and *L. plantarum* TF103-secreting α-MSH.

**Figure 4:**
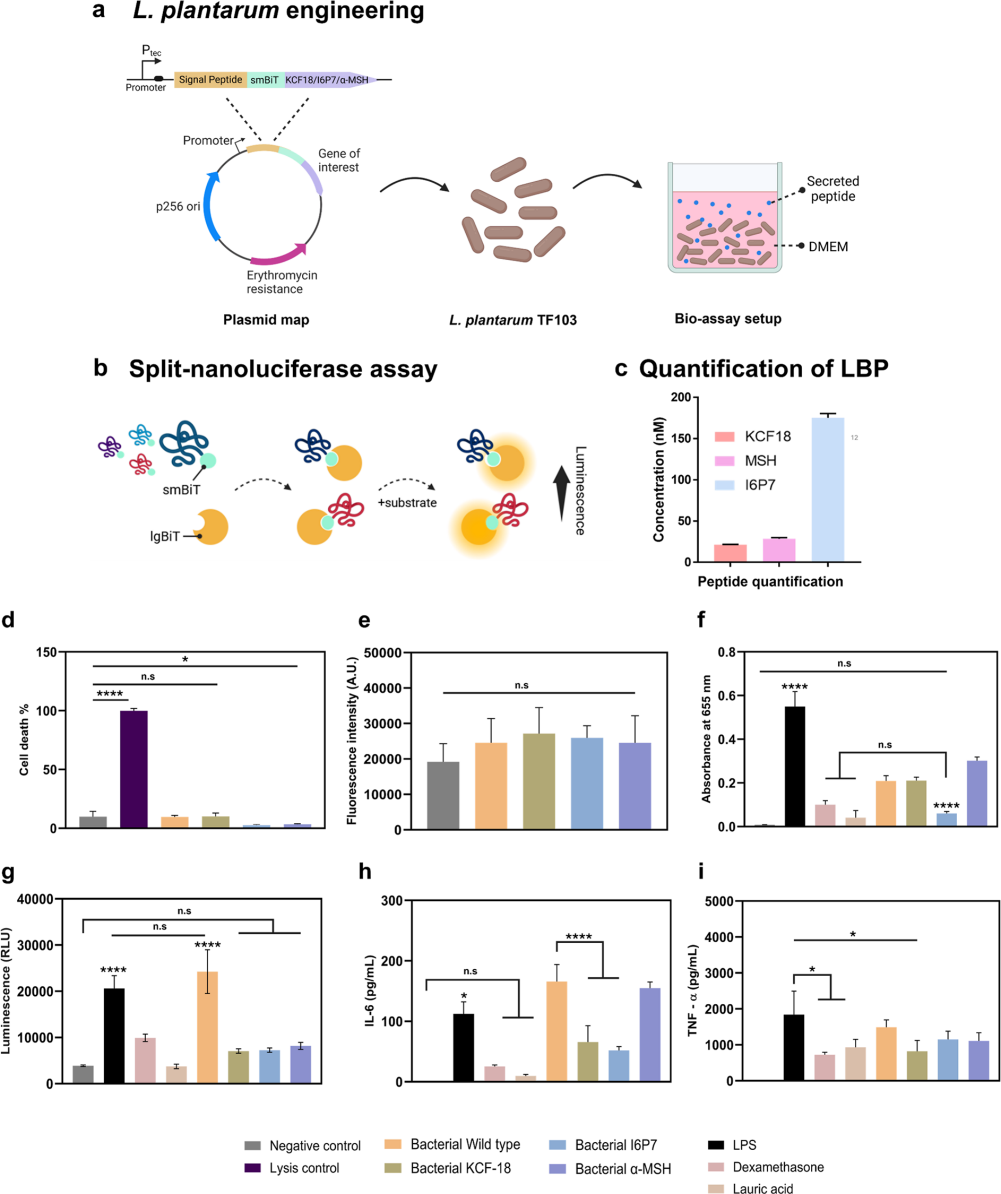
Engineered *L. plantarum* secreting KCF-18, I6P7, and α-MSH have anti-inflammatory effects. (a) Scheme of the genetic construct used to engineer the bacteria. (b) Sketch of the split luciferase assay used to quantify peptide secretion. (c) Quantification of peptide secretion from bacteria incubated in DMEM for 24 h. (d) Percentage of cell death quantified by the LDH assay. (e) Fluorescence intensity of tetrazolium-metabolizing cells via the alamarBlue assay. (f) Absorbance measured at 655 nm for the NFκB expression reporter assay via the alkaline phosphatase assay (SEAP assay). (g) Luminescence measurement for the IRF expression reporter assay via the luciferase assay. (h) Quantification of IL-6 produced by macrophages via ELISA. (i) Quantification of TNF-α produced by macrophages via ELISA. All graphs report mean ± SD, *n* = 3. Statistically significant comparisons in the ANOVA test are highlighted as * (*p*-value <0.05), ** (*p*-value <0.01), *** (*p*-value <0.001), **** (*p*-value <0.0001), and n.s (not statistically significant).

Each engineered strain was grown in MRS until the bacterial density reached 0.6 OD_600 nm_. Then, bacterial cells (roughly 3 × 10^9^ CFU/mL) were spun down, washed, and resuspended in DMEM for 24 h. The secretion of the peptides after 24 h in DMEM was quantified by using the split luciferase assay (Figure 4c). Results showed that *L. plantarum* TF103-secreting KCF-18 produced 19.5 ± 0.3 nM, *L. plantarum* TF103-secreting I6P7 produced 173.1 ± 5.4 nM, and *L. plantarum* TF103-secreting α-MSH produced 26.6 ± 1.4 nM. Although these values were lower than the lowest concentrations of the purified peptides (∼1 μM) used to develop our *in vitro* model for endotoxemia, we chose not to prolong the duration of secretion since the bacteria would likely consume nutrients such as glucose and release secondary metabolites with longer cultures.

### Effects of Engineered *L. plantarum* TF103-Secreting KCF-18, I6P7, and α-MSH on the *In Vitro* Model of Endotoxemia

We used the 24 h supernatants from the engineered bacteria to assess their potential anti-inflammatory effects using our *in vitro* model of endotoxemia. First, we studied the possible cytotoxic effects of the supernatants (Figure 4d,e) as we previously did with the peptides. We observed that the percentage of apoptotic cells was low and comparable to that of the negative control (Figure 4d). The metabolic activity of the macrophages was also similar among conditions and comparable to that of the negative control (Figure 4e). This indicates that the supernatants from the wild-type bacteria and the engineered bacteria were cytocompatible.

Then, we investigated whether treatment with the bacterial supernatants would up- or downregulate the expression of NFκB or IRF in our model (Figure 4f,g). The pretreatment for 2 h and treatment with the bacterial supernatants for 24 h resulted in a reduction in the expression of NFκB and IRF transcription factors. Interestingly, supernatants of the wild-type strain resulted in a downregulation of the expression of NF-κB but an upregulation of IRF expression. This suggests that secondary metabolites present in the supernatant of this strain positively affect the MAPK/Akt pathway that controls the translocation of the NF-κB complex into the nucleus but has no effect in the signaling of IRF pathway, which involves IFN production. The anti-inflammatory effects of other lactic acid bacteria strains have been previously shown to potentially inhibit the MAPK pathway in macrophages.^63,64^ Kang et al. investigated the anti-inflammatory effects of 75 heat-killed lactic acid bacterial strains and detected 6 strains with marked NO inhibition in RAW264.7 macrophages, among them, three *L. plantarum* strains.^64^

The effect of KCF-18 and α-MSH supernatants on the downregulation of the expression of NFκB was similar to that observed for the wild-type supernatant. This could indicate that either the secretion achieved at 24 h from the bacteria (∼20 nM for KCF-18 and ∼30 nM for α-MSH) was not sufficient or the anti-inflammatory effects of the wild-type supernatant itself might mask the effects of the peptides. The amount of I6P7 secreted by the bacteria (∼170 nM) was sufficient to show a higher downregulation of NFκB expression compared with the wild-type supernatant. For the downregulation of IRF, we observed a marked reduction in the expression of IRF for all three peptides at the same concentrations, which might indicate that the peptides have a marked effect on the IRF pathway and that this effect was not masked by the effects observed for the wild-type supernatant.

We also quantified IL-6 and TNF-α production (Figure 4h,i). Secreted KCF-18 and I6P7 significantly inhibited the production of the two proinflammatory cytokines, whereas secreted α-MSH decreased the production of TNF-α but not of IL-6, compared to LPS-treated samples and wild-type supernatants. We also quantified the percentage of M2 macrophages by determining the proportion of CD206 positive cells (Figure 5). We observed that the number of cells quantified was similar for all conditions, as expected (Figure 4d,e). From the images, we observed that all of the supernatants had a positive effect on the number of anti-inflammatory M2 macrophages. Wild-type supernatants again exhibited anti-inflammatory effects similar to secreted I6P7 and α-MSH. Interestingly, KCF-18 showed effects similar to those of our positive controls, dexamethasone and lauric acid. It is also noteworthy that the percentage of CD206 positive cells was higher for the peptides secreted by the engineered bacteria (Figure 5) compared to the pure peptides (Figure 3), even at much lower concentrations (20–100 nM vs 1 μM, respectively). This suggests a potential synergistic effect between the intrinsic anti-inflammatory effects of *L. plantarum* and the peptides, which might lower the concentration required to have a positive therapeutic effect. Similarly, the use of this model of endotoxemia could be optimized by using patient-specific cells to assess the specific host response to the bacterial strain; therefore, the strain with the highest synergy and lowest host response could be preselected for treatment. As a proof-of-concept, we took the protocol for our endotoxemia model that was optimized for murine macrophages and applied it to human macrophages (Figure S6). For this, we cultured human monocytes (THP-1 cells) and differentiated them to macrophages via standard treatment with 12-*O*-tetradecanoylphorbol-13-acetate for 48 h.^34^ Then, the human macrophages were pretreated with the bacterial KCF-18, I6P7, or α-MSH and quantified the production of key cytokines such as IL-6 and TNF-α (Figure S6b,c). The addition of the peptides secreted by the engineered bacteria lowered the secretion of both cytokines compared to LPS treatment. Positive controls with dexamethasone and lauric acid also reduced the secretion of IL-6 and TNF-α, although lauric acid did not reduce the secretion of TNF-α as much as dexamethasone did. This shows that the model of endotoxemia presented here can be easily applied to human macrophages.

**Figure 5:**
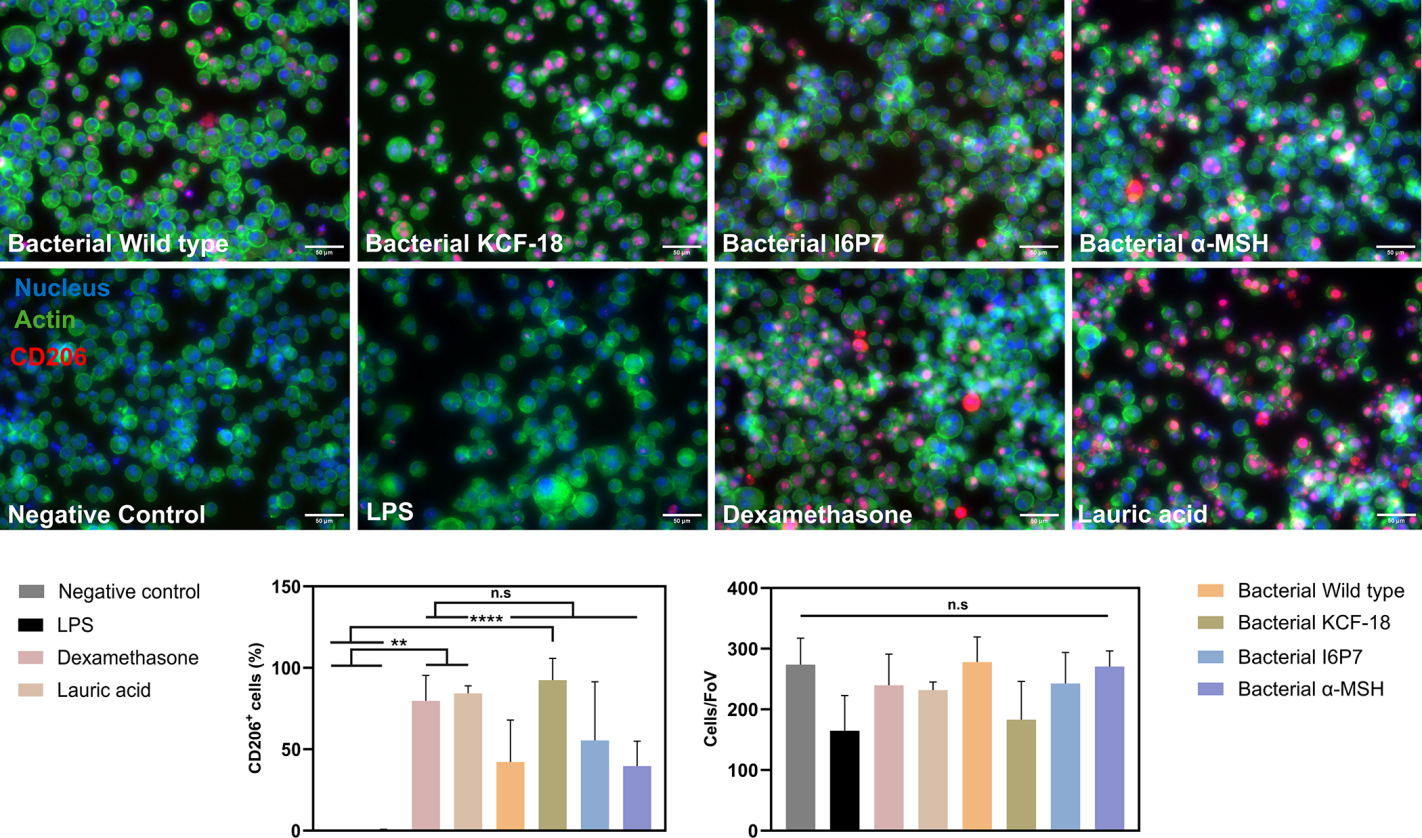
M2 macrophages quantified as CD206+ cells. Macrophages were treated with supernatants of secreted KCF-18, I6P7, and α-MSH (named bacterial KCF-18, I6P7, and α-MSH). Controls were LPS-treatment, dexamethasone-treatment, lauric acid-treatment, or no treatment (negative control) (scale bar 50 μm, blue: nuclei, green: actin, red: CD206). Left graph: percentage of CD206+ cells quantified from the images (mean ± SD, *n* = 10 images/condition); right graph: number of cells per field of view (FoV) quantified from the images (mean ± SD, *n* = 10 images/condition).

## Conclusions

This work presents a refined *in vitro* model of endotoxemia that was used to investigate the anti-inflammatory effects of three peptides: KCF-18, I6P7, and α-MSH. The cytocompatibility of the peptides was demonstrated, and their effects at different concentrations were investigated, showing inhibition of MAPK/IRF pathways, resulting in a phenotypic shift on macrophages from M1 proinflammatory to a more M2 anti-inflammatory phenotype. Then, we engineered the lactic acid bacterial strain *L. plantarum* TF103 to produce each peptide as a model of Living therapeutics and investigated the potential anti-inflammatory effects with our *in vitro* model. Supernatants from wild-type bacteria inhibited the NF-κB transcription complex, and the bacterial supernatants containing relatively low amounts of the peptides showed increased anti-inflammatory effects, pointing to potential synergistic effects of the peptides with the intrinsic anti-inflammatory properties of the bacteria. Finally, we applied the *in vitro* model of endotoxemia to human macrophages and observed similar results when treating the cells with the bacterial supernatants containing KCF-18, I6P7, and α-MSH.

## Supplementary Material



## Notes

Table S1. Concentrations of peptides used in the study. Figure S1. LPS-induced macrophage activation and NF-κB responses. Figure S2. Cytotoxicity assessment at different concentrations. Figure S3. No pretreatment followed by 24 h treatment after LPS stimulation. Figure S4. 1 h pretreatment followed by 24 h treatment after LPS stimulation. Figure S5. 2 h pretreatment with no further treatment after LPS stimulation. Figure S6. Human macrophages respond to treatment with bacterial peptides (PDF)

## Abbreviations

α-MSH: α-Melanocyte stimulating hormone
TNF-α: Tumor necrosis factor-α
IL-1β: Interleukin-1β
IL-6: Interleukin-6
MC1R: Melanocortin 1 receptor
DMSO: Dimethyl sulfoxide
DMEM: Dulbecco’s modified Eagle’s medium
SEAP: Secreted embryonic alkaline phosphatase
IFN-β: Interferon beta
NF-κB: Nuclear factor kappa B
LDH: Lactate dehydrogenase assay
PBS: Phosphate buffer saline
TPA: 12-*O*-tetradecanoylphorbol-13-acetate34
LPS: Lipopolysaccharide
ELISA: Enzyme-linked immunosorbent assay
DAPI: 4′,6-Diamidino-2-phenylindole
FoV: Field of view
MRS: Man, Rogosa, and Sharpe
OD: Optical density
SD: Standard deviation
ANOVA: Analysis of variance
MAPK: Mitogen-activated protein kinase
DM: Dexamethasone
LA: Lauric acid
