# Supplementary material for: Developing an In Vitro Model of Endotoxemia to Assess the Immunomodulatory Effects of Anti-Inflammatory Peptide-Secreting Living Therapeutics
Source: ACS Pharmacol Transl Sci. 2025 Jun 30;8(7):2180–91. doi: 10.1021/acsptsci.5c00216 (PMC12261216; doi:10.1021/acsptsci.5c00216)
Supplement: Supplementary file 1 [file pt5c00216_si_001.pdf]

Supplementary Material

Developing an *in vitro* model of endotoxemia to assess the immunomodulatory effects of anti-inflammatory peptide-secreting living therapeutics

Ketaki Deshpande[1,2], Varun Sai Tadimarri[1,2], Juliette Ramirez-Rangel[1], Shrikrishnan Sankaran[1], Sara Trujillo[1]*

[1] INM - Leibniz Institute for New Materials, Saarland University, Campus D2 2, 66123 Saarbrücken, Germany

[2] Saarland University, 66123 Saarbrücken, Germany

*E-mail: sara.trujillomunoz@leibniz-inm.de

Supporting Information

Table S 1: Concentrations of anti-inflammatory peptides (KCF-18, I6P7, and α-MSH) used in the study. S-2

Figure S 1. LPS-induced macrophage activation and NF-κB responses....................S-2

Figure S 2. Cytotoxicity assessment at different concentrations using LDH assay....................S-3

Figure S 3. No pre-treatment followed by 24 h treatment after LPS stimulation....................S-3

Figure S 4. 1h pre-treatment followed by 24 h treatment after LPS stimulation. S-4

Figure S 5. 2 h pre-treatment with no further treatment after LPS stimulation. S-5

Figure S 6. Anti-inflammatory effects of Engineered L. plantarum secreting KCF-18, I6P7 and α-MSH on human macrophages S-5

Table S 1: Concentrations of anti-inflammatory peptides (KCF-18, I6P7, and α-MSH) used in the study.

**Peptides**	**KCF-18**	**I6P7**	**α-MSH**
**Molecular weight**	2.3 kDa	1 kDa	1.623 kDa
**Stock Solution**	217 μM	500 μM	315 μM (512 μg/mL)
**Solvent used**	Filtered milli-Q water	1000 μL milli-Q, 500 μL DMSO, 500 μL Ethanol	Filtered milli-Q water
**Concentration used**	1 μM, 2 μM, and 5 μM	1 μM, 2 μM, and 5 μM	1 μM, 2 μM, and 5 μM

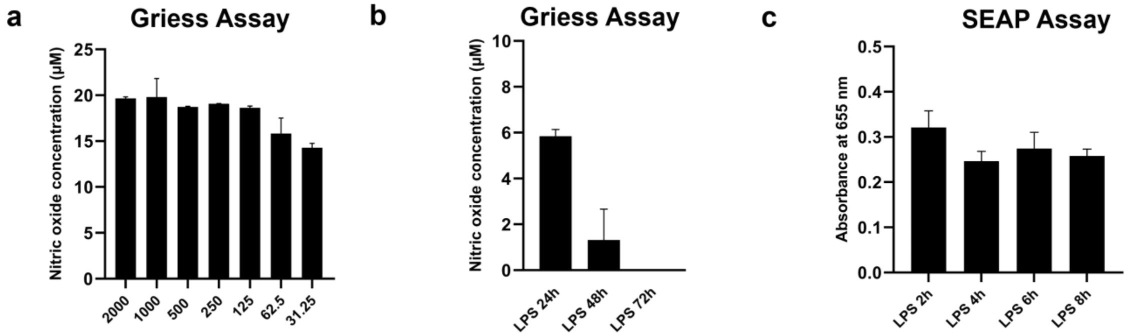

Figure S 1. LPS-induced macrophage activation and NF-κB responses. (a) Dose-response investigation of LPS to stimulate macrophages for 24h and nitrite production via Griess assay. (b) Nitrite production at different time intervals via Griess assay. (c) Absorbance measured at 655 nm for the NF-κB expression reporter assay via alkaline phosphatase assay (SEAP assay).

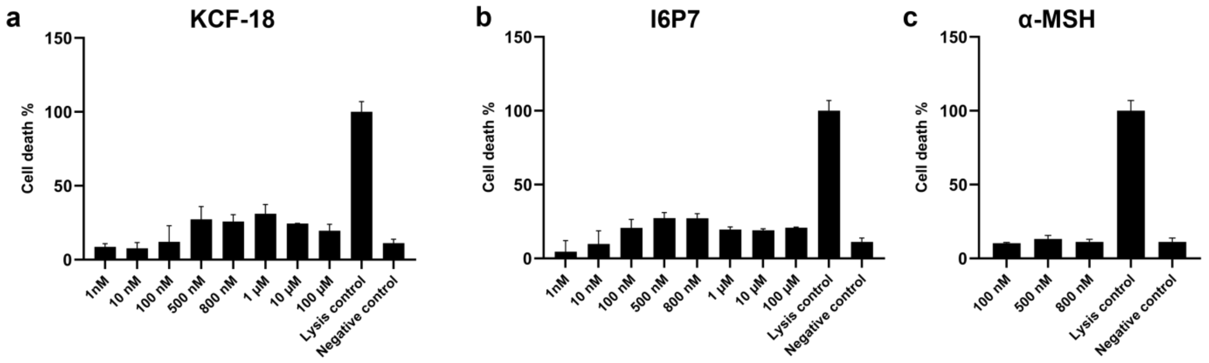

Figure S 2. Cytotoxicity assessment at different concentrations using LDH assay. (a) KCF-18 (b), I6P7 and (c) α-MSH.

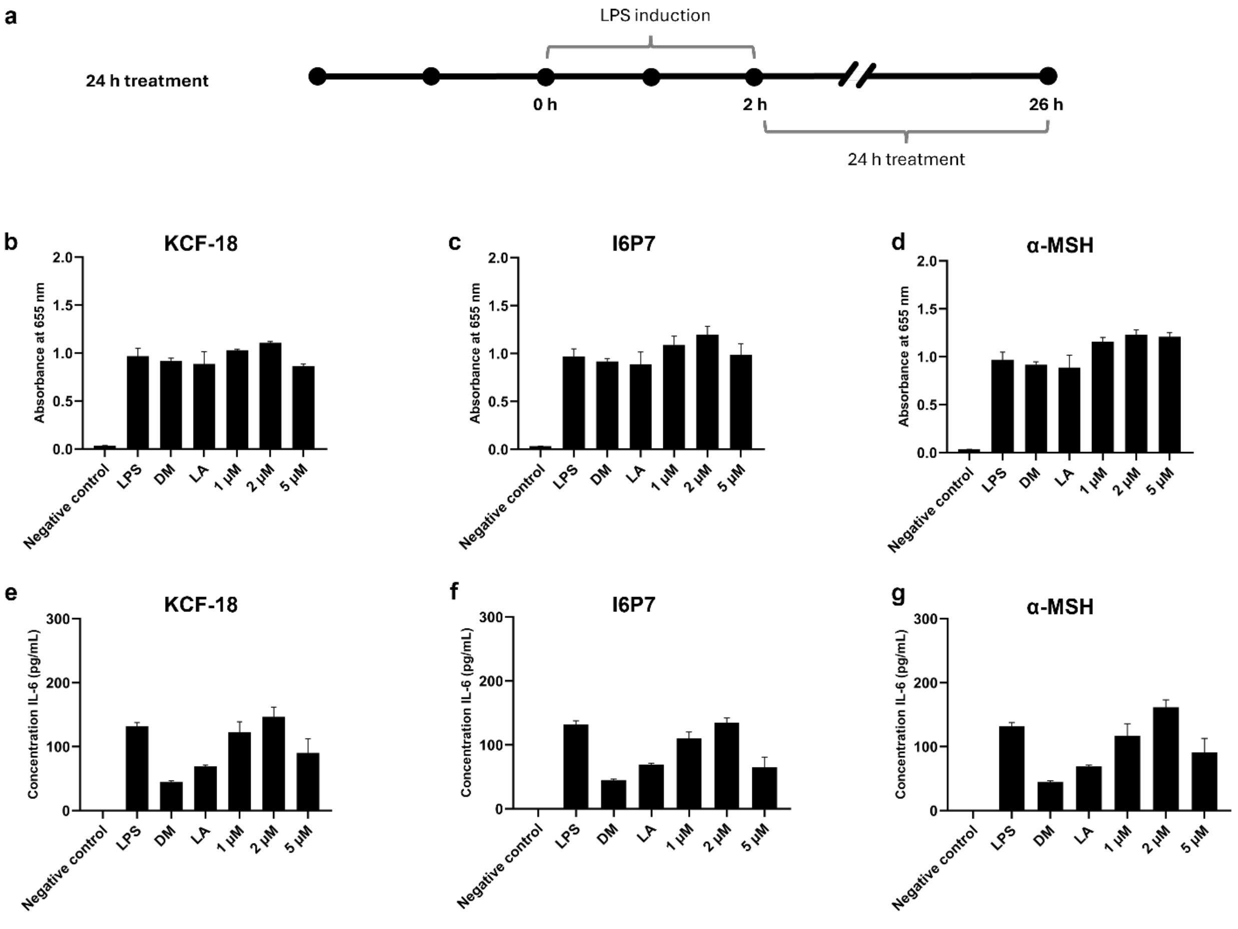

Figure S 3. No pre-treatment followed by 24 h treatment after LPS stimulation. Results for (a) Sketch of the treatment protocol for the in vitro model of endotoxemia (b-d) SEAP assay (NF-κB expression reporter assay) and (e-g) IL-6 quantification via ELISA after treatment.

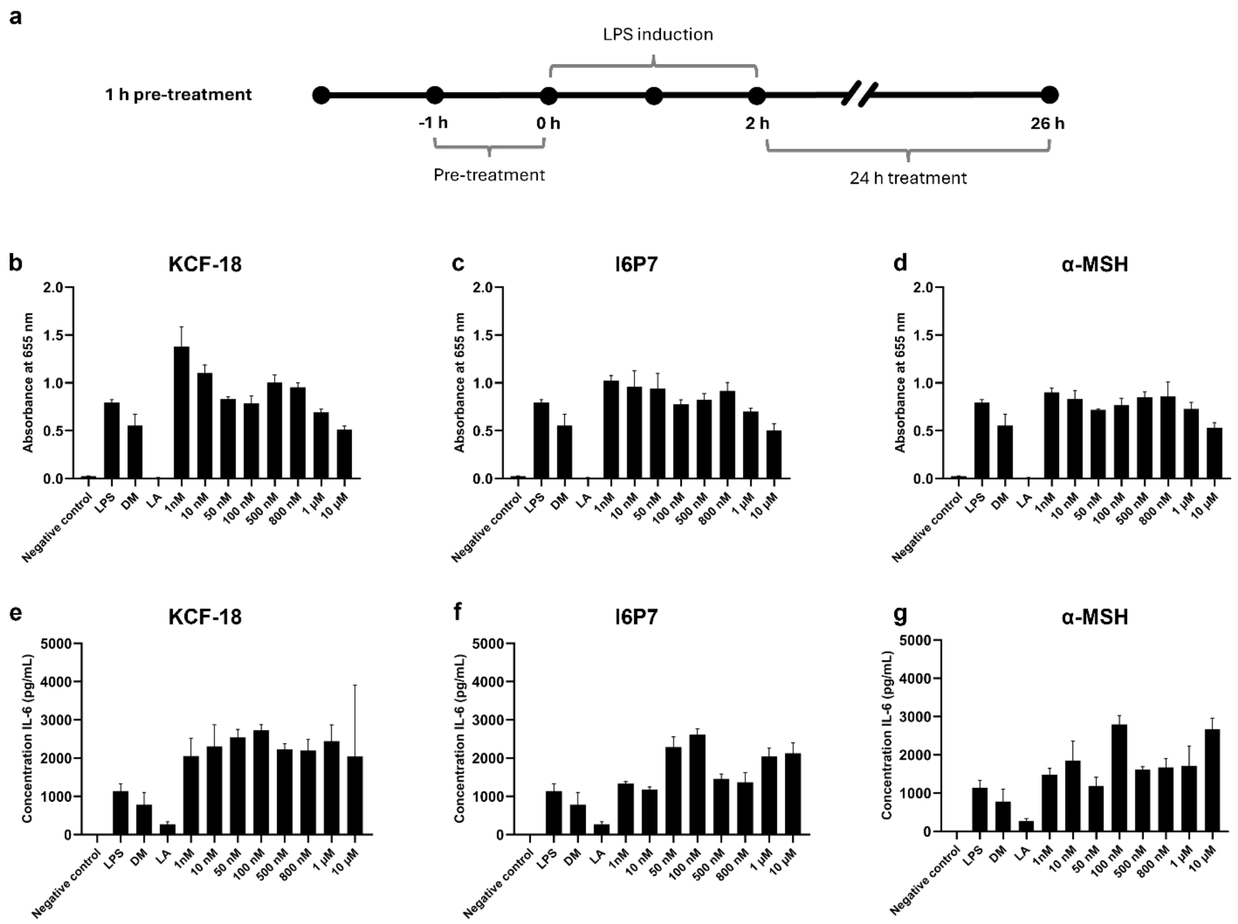

Figure S 4. 1h pre-treatment followed by 24 h treatment after LPS stimulation. Results for (a) Sketch of the treatment protocol for the in vitro model of endotoxemia (b-d) SEAP assay (NF-κB expression reporter assay) and (e-g) IL-6 quantification via ELISA after treatment.

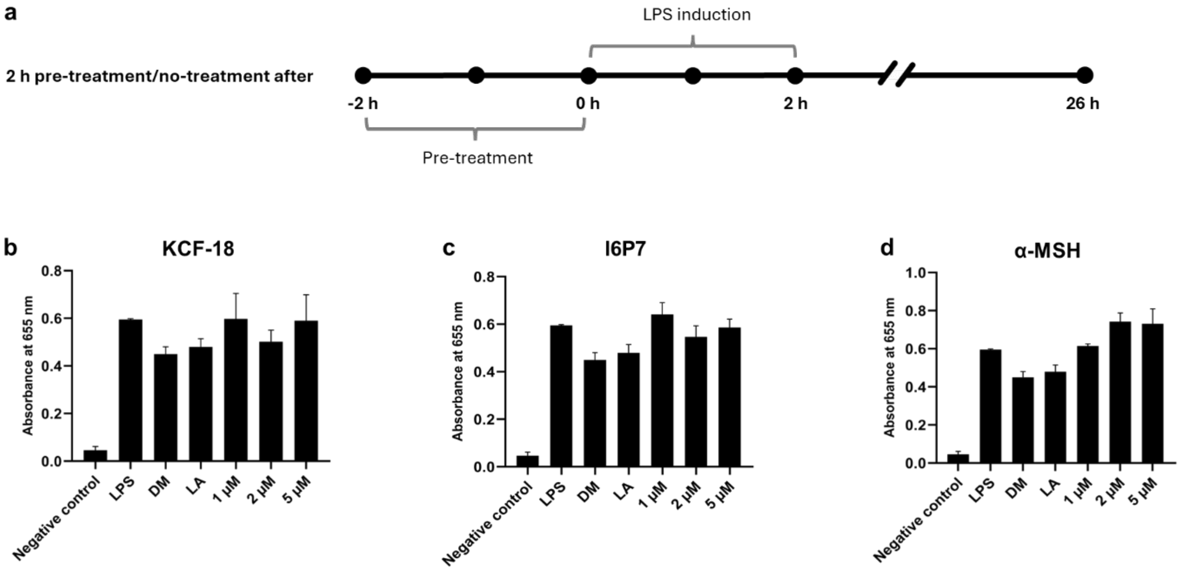

Figure S 5. 2 h pre-treatment with no further treatment after LPS stimulation. Results for (a) Sketch of the treatment protocol for the in vitro model of endotoxemia (b-d) SEAP assay (NF-κB expression reporter assay).

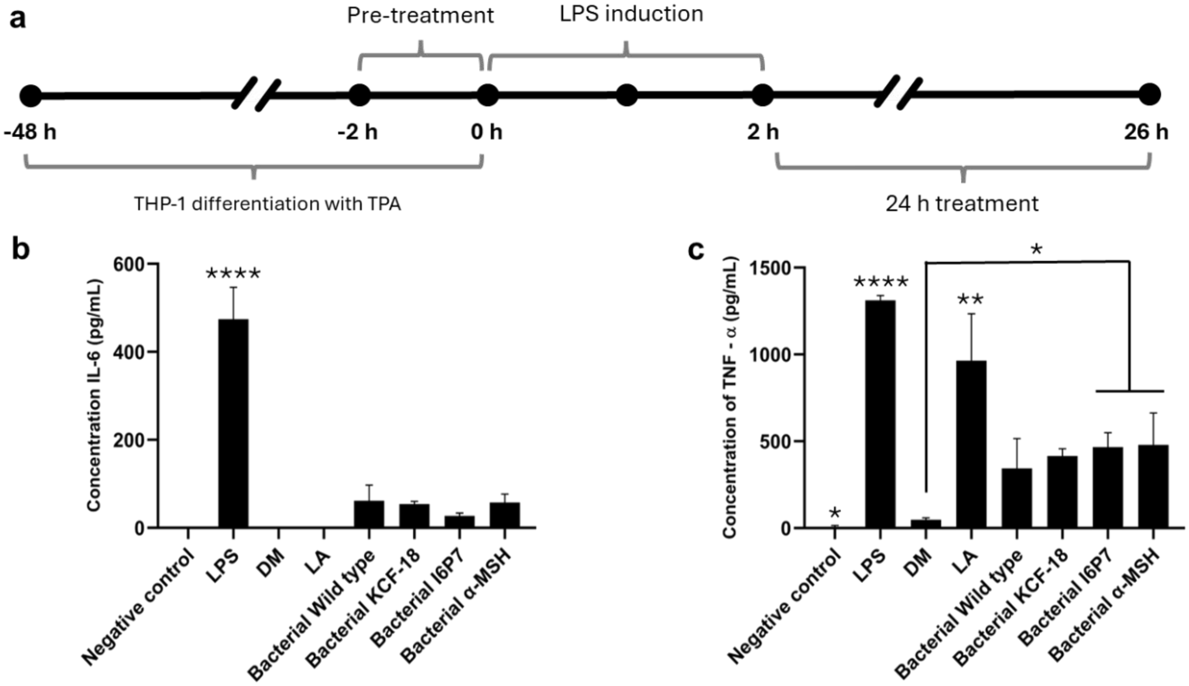

Figure S 6. Human macrophages respond to treatment with bacterial peptides. Results for (a) Sketch of the treatment protocol for the in vitro model of endotoxemia with human macrophages. (b, c) IL-6 and TNF-α quantification via ELISA after treatment.
